# Goals and expectations of older persons recovering from a hip fracture during geriatric rehabilitation: a qualitative study

**DOI:** 10.1007/s41999-026-01405-1

**Published:** 2026-01-17

**Authors:** G. F. Mattiazzo, Y. M. Drewes, P. J. M. van de Velde, A. H. Ph. Niggebrugge, M. van Eijk, W. P. Achterberg

**Affiliations:** 1 Department of Public Health and Primary Care, Leiden University Medical Center, Leiden, The Netherlands; 2 LUMC Center for Medicine for Older People (LCO), Leiden University Medical Center, Leiden, The Netherlands; 3 Department of Internal Medicine, Section Gerontology and Geriatrics, Leiden University Medical Center, Leiden, The Netherlands; 4 University Network for the Care Sector Zuid-Holland, Leiden University Medical Center, Leiden, The Netherlands; 5 Department Internal Medicine Section Geriatrics, Haaglanden Medisch Centrum, The Hague, The Netherlands

**Keywords:** Geriatric rehabilitation, Hip fracture, Qualitative research, Goals and expectation, ICF

## Abstract

**Purpose:**

Little is known about the expectations of patients with hip fracture regarding geriatric rehabilitation. We aimed to identify goals and expectations of older people regarding geriatric rehabilitation after a hip fracture in geriatric rehabilitation facilities.

**Methods:**

A qualitative study of 20 community-dwelling older adults, who all underwent rehabilitation after hip fracture in skilled nursing facilities, was conducted using semi-structured interviews by phone. Data were analyzed using thematic content analysis. The themes were additionally arranged using the International Classification of Functioning, Disability and Health (ICF).

**Results:**

The goals focused on the ICF key components of activity and participation and were described as returning to pre-fracture mobility, regaining independence in (I)ADL, returning to pre-fracture residence, and social and meaningful interactions. Expectations were related to environmental and personal factors. Environmental factors included aspects related to the care provided in rehabilitation facilities as well as the influence of informal caregivers. Personal factors focused on coping, adaptation skills, and self-efficacy.

**Conclusion:**

Patients undergoing rehabilitation have clear goals and expectations for their successful recovery. Meaningful interactions and support from the rehabilitation facility are important factors that can influence the recovery process. A patient-centered approach can be developed during goal setting by involving the patient and integrating their goals and expectations within the ICF model. This strategy maximizes the effectiveness in achieving these goals.

*Trial register and date of registration* NL7491 04-02-2019.

## Key summary points

**Aim:**

To identify the goals and expectations of older people who all underwent rehabilitation after hip fracture in geriatric rehabilitation.

**Findings:**

Patients undergoing rehabilitation have clear goals and expectations for their successful recovery.

**Message:**

A patient-centered approach can be developed during goal setting by involving the patient and integrating their goals and expectations within the ICF model.

## Introduction

The total number of hip fractures is increasing globally due to increasing life expectancy [1]. In 2019, the age-standardized incidence rate of hip fractures was 182.5 (141.9–230.9) per 100,000 individuals [2, 3]. The impact of hip fractures is huge, with a mortality rate of 25% in the first year [4, 5]. In addition, hip fractures are associated with a rise in disability, with 40% of the survivors failing to regain mobility at 1-year post-fracture. Moreover, 60–80% of patients still have limitations in activities of daily living (ADL) after returning home [6, 7]. A hip fracture not only affects the physical condition, but also leads to psychosocial limitations and has a strong impact on quality of life [8]. Patients experience difficulties regaining control and independence. Having to accept support that they did not previously need can be perceived as a threat to their independence [9].

Studies have shown that involving patients in their own therapy results in an increase in autonomy and functional outcomes [10]. Motivation in the form of positive thinking can lead to adaptability to new situations and the ability to readjust lives by using technological support. [11]. Consequently, stimulating motivation by achieving self-sufficiency and meeting functional needs are an essential aspect of rehabilitation [12]. This can be achieved through goal setting. A Cochrane review on the role of structured goal setting showed an association between goal setting and higher levels of motivation, self-efficacy, and health-related quality of life [13]. The most prominent aspects of goal setting regarding recovery among community-dwelling older patients with a hip fracture are long-term expectations regarding mobility, ADL, pain, and fear of falling [8, 12, 14].

In the Netherlands, 55% of patients with a hip fracture are discharged from hospital to geriatric rehabilitation [15]. Geriatric rehabilitation is provided in skilled nursing homes that aim to restore physical function or enhance residual functional capacity and participation in older patients using a multidisciplinary team approach. These nursing homes are encouraged to set goals to facilitate personalized and integrated care [16]. Previous studies have examined the goals of patients with a hip fracture in the acute care setting. Fixed goals were set by healthcare professionals to assess the patients’ progress [17–19], while one qualitative study looked at goals from the patient’s point of view [18]. Currently, to our knowledge, there are few studies that explore the goals and expectations of patients with a hip fracture who followed a rehabilitation program in the Netherlands [11, 20]. Therefore, the aim of this qualitative study was to identify and explore the goals and expectations in achieving the predetermined goals of older people during geriatric rehabilitation after a hip fracture in rehabilitation facilities, especially by focusing on the patient’s expectations for assistance and guidance in achieving their goals. Healthcare professionals can use this knowledge to design more personalized rehabilitation programs tailored to the needs of patients recovering from a hip fracture.

## Methods

### Study design

This qualitative study is embedded in the inception cohort study HIP CARE: Inventarisation of Prognostic factors and their Contribution toward Rehabilitation in older Persons. HIP CARE investigates which factors have a positive impact on geriatric rehabilitation after a hip fracture [21, 22]. For this qualitative study, we explored patients' experiences regarding goals and expectations during rehabilitation. Due to the COVID-19 pandemic, we conducted telephone interviews with participants. We used the COREQ checklist for the design of the study and reporting of the data [23].

### Recruiting participants

Participants in the HIP CARE study were older persons with a hip fracture who were admitted to the acute care unit of the Haaglanden Medical Center (HMC +) in The Hague. They were previously community-dwelling patients, aged 70 years or older with a unilateral hip fracture who were eligible for (inpatient) geriatric rehabilitation. Exclusion criteria were: previously residing in nursing homes, being younger than 70 years of age, having a pathological hip fracture, being unwilling or unable to give informed consent, having insufficient Dutch language skills, and already being included in this study due to a prior hip fracture.

Participants in the HIP CARE study who were discharged from rehabilitation up to 6 months received an information letter by mail informing them about the qualitative study and inviting them to participate in a telephone interview. The researchers contacted the participant 1 week later to provide further information and to obtain verbal consent. An appointment was then made for the interview. Initially, 20 patients were invited, 12 of whom agreed to participate. To increase the number of participants, we consecutively sampled 11 patients who had been discharged from geriatric rehabilitation between 6 months and 1 year. To ensure that these participants did not have cognitive disorders affecting their memory, we selected patients with a score < 10 on the six-item cognitive impairment test (6-item CIT, maximum score 28; a score ≥ 10 indicates cognitive impairment) [24]. A further eight participants were then included. The reasons for refusing to participate were either that a telephone interview was too strenuous (n = 6) or that a family member had advised against it (n = 5). Participants gave verbal consent at the start of the interview. After the COVID-19 restrictions were lifted in June 2020, signed informed consent was obtained.

### Data collection

Two researchers, a medical doctor (GM) and a master's student (PvdV), who were trained in qualitative research, conducted semi-structured interviews in Dutch by telephone with a median duration of 17 min (range10–30 min) between April 22 and June 9, 2020. They worked in a research team with extensive experience in qualitative research and research with older persons. Healthcare professionals, patients, and caregivers often differ on how they define goals. This variation was explicitly considered during the study’s preparatory phase. To ensure the relevance and clarity of the interview guide, the research team drew on the expertise of two physicians actively working in geriatric rehabilitation to formulate the questions for participants. Field notes were taken during the interviews. The interview guide included topics such as goals and expectations during and after geriatric rehabilitation. The systematically structured interviews covered the period from admission to geriatric rehabilitation to discharge home. Additional questions were asked when a deeper understanding was needed. All interviews were included in the final analysis. Interviews were recorded and transcribed verbatim by the interviewers or a professional transcriber.

### Data analysis

Data analysis was carried out by means of thematic content analysis [25]. The first five interviews were listened to repeatedly by two researchers (PvdV and GM) to familiarize themselves with the data. The two researchers used these first five interviews to create a coding tree. The interviews were analyzed line by line using an open coding system. Subsequently, the inductively derived codes were rearranged into clusters for axial coding. This was discussed in the research team until consensus was reached. The two researchers independently coded the remaining 15 interviews to increase reliability. New codes were added when necessary. These new codes and discrepancies were also discussed until consensus was reached. After coding, the key issues were organized into themes. The research team discussed the themes that emerged from the interviews. Comparison with existing theories revealed that topics with associated key themes could be organized according to the International Classification of Functioning, Disability, and Health (ICF) framework [26]. The ICF model is a standardized framework for describing functioning in terms of five key components: body functions and structures, activity, participation, environmental factors, and personal factors. [27]. In the current analysis, activity was limited to person-oriented activities, while participation was interpreted as social functioning in society and IADL. Environmental factors were related to the assistance needed to achieve their goals, while personal factors dealt with the inherent qualities each individual required to achieve their predetermined goals [28]. Based on this ICF framework, the coding scheme was enriched, and all interviews were reread by GM to explore related concepts. Finally, the analysis resulted in a structured overview of the goals and expectations of older people, arranged according to the ICF framework. All interviews were analyzed in Atlas.ti 23. Quotations were selected and later translated into English by a native speaker.

### Ethical considerations

The HIP CARE study was approved by the Medical Ethics Committee Southwest Holland (protocol number 18-081 NL66871.098.18) and published in the Netherlands Trial Registry (NTR) (trial registration number NL7491).

## Results

The 20 participants were community-dwelling older adults who were admitted to the geriatric rehabilitation unit for rehabilitation after hip fracture surgery. Their mean age was 82 years (range 71–95 years), 35% were male,and 65% were female. Prior to the hip fracture, 18 of the 20 participants were ADL independent, while 4 participants required an assistive device for indoor mobility. The demographic characteristics of the participants are summarized in Table 1.

**Table 1:** Demographic characteristic of the participants

Participant number	Gender	Age (years)	Having an Informal caregiver	Pre-fracture	
				ADL	Mobility aid (indoors)
1	Male	77	No	Independent	No
2	Female	80	Yes	Independent	No
3	Female	88	No	Independent	Yes
4	Female	81	No	Independent	No
5	Female	86	No	Independent	No
6	Male	72	Yes	Independent	No
7	Female	76	Yes	Independent	No
8	Male	77	No	Independent	No
9	Female	78	Yes	Independent	No
10	Female	88	No	Independent	Yes
11	Female	90	Yes	Independent	No
12	Female	84	No	Home care	Yes
13	Male	71	Yes	Independent	No
14	Male	95	No	Home care	No
15	Female	75	No	Independent	No
16	Female	80	Yes	Independent	No
17	Male	74	Yes	Independent	No
18	Male	79	Yes	Independent	No
19	Female	92	Yes	Independent	Yes
20	Female	91	No	Independent	No

**Fig. 1:**
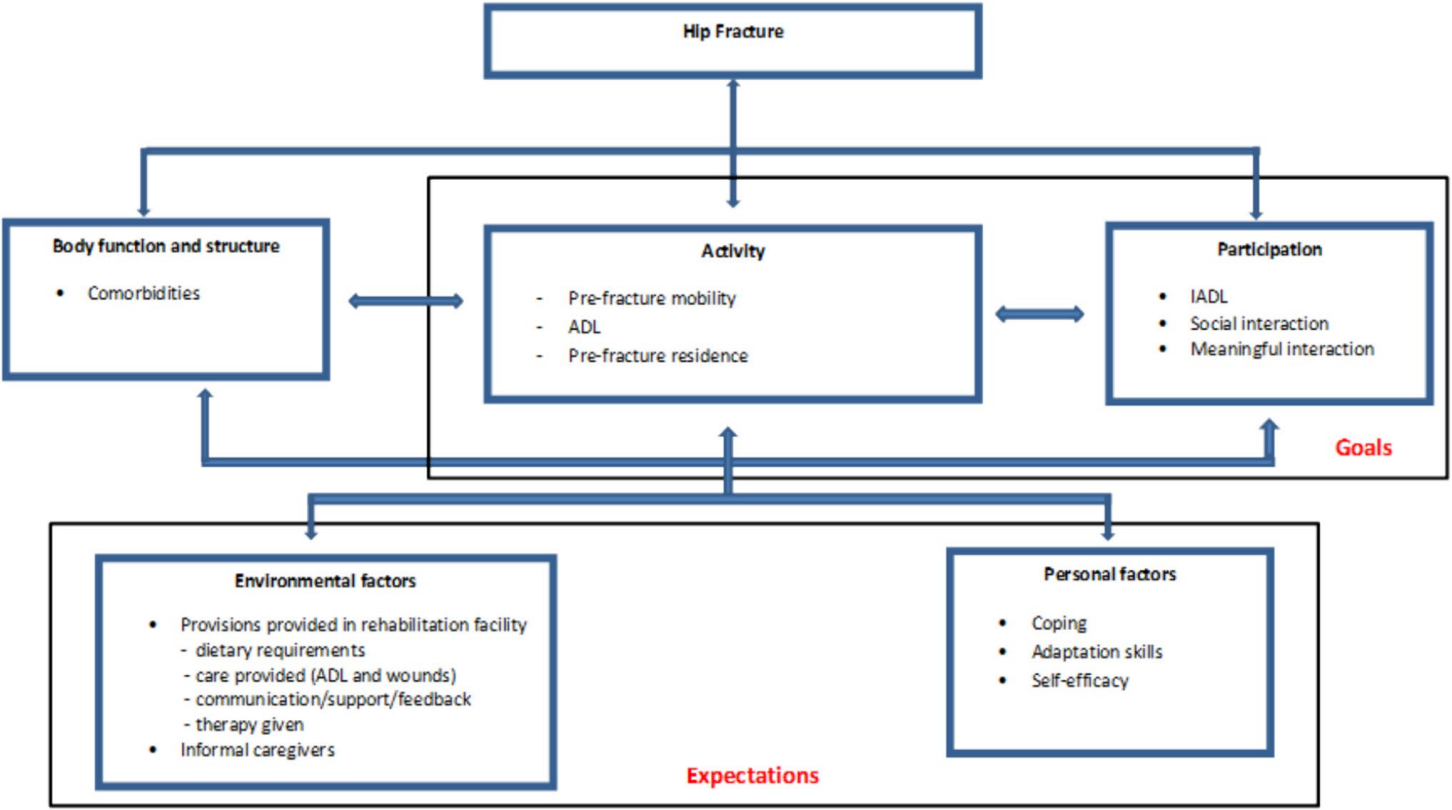
Goals and expectations classified according to the ICF framework

The goals of the patients appeared to focus on the ICF key components of activity and participation (Fig. 1). Goals categorized as activity were goals related to pre-fracture mobility, ADL, and pre-fracture residence, while goals regarding participation focused on IADL, social interaction, and meaningful interaction. According to the ICF key components, patients’ expectations were categorized as environmental and personal factors. The care provided within rehabilitation centers, along with the support of caregivers, constituted the primary environmental factors. In contrast, self-efficacy, coping strategies, and adaptive skills were identified as personal factors. Patients perceived both personal and environmental factors as contributing to their ability to achieve their rehabilitation goals.

### Goals: activity

#### Pre-fracture mobility

Most of the participants led an independent life prior to the hip fracture. All of them were mobile before the fracture, some with the help of a mobility aid. The hip fracture and subsequent surgery made all the participants dependent on healthcare professionals, resulting in a sense of loss of autonomy. Therefore, regaining mobility during the geriatric rehabilitation was their primary goal. According to the participants, they expected to be able to return to their previous way of living by regaining their pre-fracture mobility. This was demonstrated by the statement of one participant.

*Yes, I definitely had clear goals for what I wanted to achieve, to be able to walk again like I did before. (P5)*

Some participants gave a comprehensive description of mobility. They focused not only on walking, but also on what they could do once they were mobile again. They defined this as being able to climb stairs and go outside. This would give them the freedom to move around in their home and beyond.

*I said I want to go home, I live alone in my own house, and I wanted to be able to climb stairs. And to walk the dog, and so they tried to make that happen. (P15)*

#### Activity of daily living

The need to be self-sufficient was emphasized by many participants. This was expressed in the context of ADL care. The desire to regain independence in ADL stimulated the rehabilitation process. Several participants indicated that relying on ADL professionals for self-care tasks such as dressing and bathing affected their sense of independence.

*The goal was, if I can shower myself, get out of bed on my own and I can move around by myself, then in principle, by my own standards, I can go home. (P6)*

#### Pre-fracture residence

None of the participants had been institutionalized before the hip fracture. They were older persons living in their own homes. During the geriatric rehabilitation, a focus on regaining mobility was also linked to returning to their pre-fracture residence. Returning to the pre-fracture residence was associated with being healthy again.

*I wanted to return home healthy. Back to my allotment. Walking on my own two feet again. That was the goal of rehabilitation. (P11*)

Furthermore, their home was a familiar place where daily activities took place. For them, the comforting environment of the pre-fracture home was very important*.*

*I longed to go home to my doggie, and well, all in all I spent three months there, and I thought that was the worst thing in the world. (P15)*

### Goals: participation

#### Instrumental activities of daily living

Several participants reported another aspect of independence: not only mobility and ADL, but also their desire to regain their IADL skills such as shopping, cooking, and other daily tasks. These more complex domestic and household management tasks were seen as necessary to participate in community life.

*I’m 81 years old already, but I want to stay independent and, of course, go out by myself. I want to do my own shopping and take care of everything myself. (P4)*

#### Social interactions

Participants described social interaction as an important aspect of daily life. In the interviews, several participants mentioned hobbies and sports as important forms of participation next to IADL. These activities were valuable activities which they saw as important goals of their recovery.

*I wanted to get back to doing my sports and clubs and many of the things I used to enjoy. (P5)*

#### Meaningful interaction

Relationships with others were also valued as important social interactions. Participants described wanting to improve their mobility to be able to take care of a spouse again, of their pets, or to volunteer in the community.

*Well, I’m involved in the neighborhood watch, so a couple of times a week, we go for a walk around old Rijswijk. But, of course, walking is still a problem. (P17)*

### Expectations: environmental factors

#### Provisions provided in the rehabilitation facility

Participants discussed with the interviewer how they expected to achieve their goals. The participants clarified that it was the facility's duty to assist with the rehabilitation process. A good rehabilitation program, they indicated, not only treats the hip fracture, and restores mobility, but also helps the patient set realistic expectations about how they want to achieve their goals. This involved two aspects of the ICF framework. The first aspect was activity, as expressed in the treatment of physical problems such as activities of daily living (ADLs) and wound care. The second aspect was linked to environmental factors, including guidance on nutritional requirements to manage comorbidities, feedback, and support through communication during participants' stay in the rehabilitation program, and the provision of both physical and emotional support by healthcare professionals during geriatric rehabilitation. Participants with diabetes seemed to be critical of the diet they received. In their view, a balanced diet could positively influence their rehabilitation process. In some cases, family members regularly brought appropriate meals that were not provided by the geriatric rehabilitation facility. In addition, the quantity of the food was criticized. Furthermore, the meals were not only nourishment but also a welcome diversion during their stay in geriatric rehabilitation.

*That tastes like nothing. Or it doesn’t taste good at all. And, well, some overcooked vegetables. I mean, I eat a lot healthier at home than I did there. And I lost three kilos because I just didn’t want to eat. I wanted to eat, but I couldn’t eat that food. It was absolutely unappetizing, and that’s not good for people in rehabilitation. You need to eat healthy things. (P3)*

Many participants emphasized the importance of professional support from nursing staff. According to the participants, nurses attended to somatic issues such as wound care and assisted in the early stages of ADL. Nursing staff supported participants in gradually regaining premorbid ADL. In addition, they comforted participants by attending to their personal needs, taking time to listen, and being polite during bedtime.

*At first, the care was quite intensive, but it gradually decreased, and at a certain point I was able to do it myself. So, with washing and dressing, I progressed step by step. Initially, I received full assistance, but over the course of two months, it was reduced until I could be independent. (P5)*

Before hip fracture surgery, some participants were informal caregivers. They talked about how difficult it was to deal with the diminishing ability to balance rehabilitation and caring for their partner. They regretted not receiving the counseling they needed after they were discharged. They would have liked additional instructions on how to deal with this situation. One participant made clear how much this guidance was missed.

*I expected more from it. That they would do more for people, you know? I thought aftercare would be provided by the geriatric rehabilitation. That’s not the case now. I’m a caregiver, yes. (P8)*

Some participants emphasized that communication with healthcare professionals played a significant role in rehabilitation. They described the need to be encouraged with feedback and reassurance that they were progressing, that their recovery process and progress were improving during rehabilitation.

*You would expect to get a little more feedback from a physiotherapist, right? I mean, they treat people with these kinds of fractures all day long. I'm sure recovery can vary, but there has to be some kind of average. And, well, they not really inform you. (P6)*

Interaction with the physiotherapist about the intensity and quality of therapy was often mentioned as a key factor in rehabilitation. One participant underlined the value of these conversations and how they resulted in additional exercises.

*I had regular conversations with the physician, the physiotherapist, and the occupational therapist about progress, the results achieved, and the ultimate goal. My input concerned the intensity of the training sessions and planning the duration and working towards a discharge date as soon as possible. (P18)*

When returning to their pre-fracture residence, some participants had to adjust their home. The occupational therapist provided the necessary expertise in wheelchair fitting, transfers, and home adaptations.

*The occupational therapist also came to see if I could go home. I live on the second floor, with an outside and an inside staircase. (P11)*

#### Informal caregiver

Informal caregivers were seen as an important aspect of social support. Half of the participants had an informal caregiver. Informal caregivers were an asset in the recovery process. Participants' children were able to organize additional help and thus enable a speedy discharge, while partners provided additional care after discharge when home care was not required.

*I have sons, and they made sure everything was in order in the bathroom and that the bed upstairs was set to the right height. The kids managed to get everything organized. (P19)*

### Expectations: personal factors

#### Coping

Several participants stated that, in addition to support from the environment, self-motivation was needed and had to come from within. This internal drive was needed to achieve the goals. They must rely on their own ability to achieve these goals. This aspect was noted primarily when discussing the return to pre-fracture mobility.

*But doing it yourself and working hard—that was positive. I also said, “Yes, walk on your own, do it yourself, try to get out, but you really have to put in a lot of effort yourself.”. (P7)*

Several participants who were motivated were actively involved in their treatment plan. These participants indicated the importance of their personal drive as a facilitator in regaining independence.

*I was very much involved, but I’m also not someone who accepts being excluded, so I always have to have my own input as well. (P19)*

Some participants had set a time limit for achieving their goals. They expected to be able to walk again after their discharge from geriatric rehabilitation. However, during their stay in geriatric rehabilitation, it became clear to some that not all the predetermined goals could be achieved in this short time frame. The way in which the goals were readjusted varied from participant to participant. Some struggled to accept these adjustments, while others showed flexibility by accepting changes in goals.

*Because I’m quite a down-to-earth person, and if it can't be done the way it should be done, then it must be done the way it can be done, that’s what I say. (P18)*

#### Adaptation skills

Several participants accepted adjustments to their goals. When expectations were not met, adaptation strategies were demonstrated. Three distinct scenarios were observed. The first was that additional home care was seen as an accepted means to enable returning to the pre-fracture residence. The second situation involved assisting them in regaining their independence using a mobility device.

*I’m not sure if I made that clear enough. Because eventually, you also start adjusting your own expectations. (P2)*

Third, some participants put high expectations on themselves about regaining mobility in a short period of time. This generated negative emotions such as sadness and regret. As a result, they readjusted the time frame for their goals instead of setting short-term goals.

*Yes, that [...] takes a long time, of course. I mean, I [...] heard from the physiotherapist that I would be receiving physiotherapy for a year. [...] Is that really the timeline? I never knew that. So, uh, yeah, that is a bit disappointing. (P16)*

#### Self-efficacy

Participants used different methods to achieve their goals. Participants who were more in control of their own therapy chose to set their own goals. These participants expressed a desire for mobility and therefore took advantage of every opportunity. Some participants also described how taking responsibility for their therapy could have a positive effect on the outcome. One example was the ability to influence a future discharge.

*Very good, because through intensive training, well, you obviously have to do it yourself. The therapist gives you instructions, but you have to practice on the bike, with the walking bridge, and climbing stairs. And because I practiced so much, I was able to go home a week earlier than planned. (P13)*

Others willingly accepted the goals set by healthcare professionals, choosing a more passive role during rehabilitation. According to these participants, healthcare professionals were the experts who had more experience in creating a treatment plan and setting goals. These participants described that they followed the physiotherapist who focused on regaining mobility and the occupational therapist who set IADL goals. One participant even referred to the healthcare professionals as “masters” who better understood the rehabilitation process.

*No own plan? No, of course not, I didn’t have one. I left that to the Masters. (P12)*

However, a smaller group felt that their lack of experience prevented them from being involved in the process.

*And besides, well, of course you don’t have any expertise, so you just keep quiet. The input really only comes from the other side. (P14)*

## Discussion

The aim of this study was to examine the goals and expectations of older people during geriatric rehabilitation after a hip fracture. The patients' goals were related to the ICF key components of activity and participation, while the patients' expectations appeared to be related to the ICF key components of environmental and personal factors. The goals were described as: return to pre-fracture mobility, regain independence in (I)ADL, return to pre-fracture residence, and social and meaningful interactions. The category “environmental factors” includes aspects related to the facility's ability to provide guidance on matters like dietary requirements and medical professional communication, as well as the influence of informal caregivers. Personal factors focused on self-efficacy, coping strategies, and adaptation skills.

Patients' desired outcomes after a hip fracture have been frequently investigated. Regaining mobility is a priority for all patients and all healthcare professionals [29, 30]. In addition, other goals such as regaining ADL, returning home, and improving overall health were frequently discussed during the interviews. The findings in our current study regarding goals are consistent with previous literature [20, 29]. In the ICF framework, activities are defined as the execution of a task or action by an individual, while participation is defined as involvement in a life situation [26]. These aspects formed the goals of the participants in our study. However, the ICF framework developed by the World Health Organization recognizes social interaction as a key component of participation but it does not provide a detailed description of meaningful interaction within the core aspect [26, 31]. This form of interaction is defined as personal relationships with friends, family, volunteer work and being active [32, 33]. In our study, the participants emphasized the importance of social interaction and returning to normal pre-fracture life. Activity and participation are included as rehabilitation goals in the ICF model. As a result mobility can be viewed as a tool to restore participation. Social and meaningful interaction can increase motivation for self-care [34]. This, in turn, may help healthcare professionals to create guidelines for rehabilitation by focusing on the social as well as the meaningful aspects [32].

Goals and expectations are based on personal needs [35]. Nevertheless, interviews showed an overlap in terms of goals and expectations. Studies of these patients' expectations are rare, although asking about both goals and expectations can be significant [36]. In the hospital setting, patient expectations are influenced by the surgical aspect of hip fracture such as wound healing, secondary prophylaxis for thrombosis, and pain management [37]. Our study focused on patients’ expectations when discharged to geriatric rehabilitation.

Environmental factors play a vital role in the expectations of patients with a hips fracture. The facility can influence recovery by providing factors such as dietary assistance, therapy, and even advice to informal carers. During our analysis, we found that patients considered the role of the facility in their process. The focus was not only on mobility and ADL, but also on the facility's contribution to rehabilitation. One recurring aspect was advice on nutrition. Studies show that poor nutrition can impact functioning, increase healthcare costs, and is associated with high mortality [38]. Earlier studies have shown that 4%–39.4% of patients with a hip fracture are malnourished prior to hospital admission [39]. These vulnerable older adults start with reduced nutritional levels prior to fracture, and improving nutritional therapy during rehabilitation is therefore critical. In older patients with a hip fracture, this is an important strategy to reduce mortality and postoperative complications [40].

Good collaboration between patients and healthcare professionals is beneficial and has an impact on quality of life [13, 41]. Therefore, it is important to bridge the gap between patients' expectations of their recovery process and reality [42]. Inquiring about participation may assist healthcare professionals in determining and ultimately modifying objectives to maintain meaningful and social interaction while maintaining quality of life. Timely information from healthcare professionals working in a multidisciplinary team can have an impact on patients' involvement in their own rehabilitation process [43]. Feedback can be used to understand and improve interactions between healthcare professionals and patients [35]. In addition, healthcare professionals can motivate patients through feedback on progress and counseling [30]. This, in turn, can help to manage patients’ expectations of achieving and adjusting goals [44].

Not only patients, but also informal caregivers need information about the progress of rehabilitation [43]. Most informal caregivers have little knowledge about rehabilitation after hip fracture [45]. Nevertheless, informal caregivers play an essential role during discharge and are therefore critical in influencing functional outcome [46, 47]. Informal caregivers provide a sense of security. This allows patients to focus on personal factors such as positive attitudes, optimism, expectations, and self-efficacy, which facilitate the role in recovery [48]. These factors were frequently encountered in our population. Another aspect was the adaptability of patients with a hip fracture. Sims-Gold recognized the importance of adaptation, which was required for patients with a hip fracture return to pre-fracture activities [48]. This was observed in our study with the acceptance of mobility devices. These devices provided participants with a means to return to their pre-fracture residence and daily activities.

According to Dyer et al., the ICF framework can be used to understand the patient's expectations and social context [6]. Initially, our interviews were not structured according to the official ICF codes. However, during the analysis we observed that the ICF framework facilitates a systematic approach to goal setting. This allows both patients and healthcare professionals to be effectively guided in understanding patients' goals and expectations. It visualizes not only the physical aspect of a hip fracture, but also the psychological and social components during recovery [49]. Therefore, the ICF framework should be incorporated as a standardized part of the treatment plan.

### Strengths and limitations

The strength of our study is that it was embedded in a large interception cohort study from the same hospital. Therefore, care in the acute phase was comparable. Furthermore, the analysis started in an inductive form, but throughout the analysis process the ICF framework provided a structural foundation. For the telephone interviews, an appointment was made at the convenience of the participants. The interviews were all planned on a one-to-one basis so that the participants could speak freely. Some limitations were noted. First, the interviews were performed by telephone due to the COVID pandemic. Consequently, nonverbal communication, including body language, could not be incorporated into the analysis. This limitation, combined with the relatively short interview duration (median duration of 17 min), may have constrained the depth and nuance of the findings.

Secondly, despite selecting participants on cognition to avoid recall bias, the memory of details during rehabilitation may be affected. Thirdly, only a small number of participants mentioned engaging in social interactions through volunteer work. This may be attributed to the impact of the COVID-19 pandemic that prevented them from continuing these activities, which therefore were not relevant to the interviews.

## Conclusion

This study investigated the goals and expectation of patients recovering from a hip fracture during geriatric rehabilitation. By actively involving patients and integrating their expectations into the ICF model, a patient centered approach can be established. Regaining mobility, as well as social and meaningful interactions, was the primary goal expressed by the patients. Additional, supportive assistance from the facility and informal caregivers and their own personal factors positively influence recovery. By aligning rehabilitation strategies with what patients find meaningful, the effectiveness of goal achievement can be maximized.

## Data Availability

The datasets used and/or analyzed during current study are available from the corresponding author on reasonable request.

## Appendix 1: Topic list and example questions

After transcribing the first six interviews, we discussed whether questions were missing or needed adjustments. Questions that were added or adjusted are marked with (*). After performing my first interview, a second adjustment was made marked with (**).

**Table:**

Introduction	Patient and researcher introduce themselves on the recording, clearly stating consent for the interviews Brief introduction with an explanation of what the procedure will be **Start recording** *As mentioned, I would like to ask you a few questions about the rehabilitation phase. There are no right or wrong answers—we are interested in your experiences, and this information is very valuable to us* *I expect this conversation will take about half an hour. As discussed, an audio recording of the interview will be made. Once the recording has started, I will ask you again for your consent to participate in the interview and to have it recorded* *Before we begin, do you have any questions about the interview?* *If you don’t have any further questions, I would like to start the interview. I will now start the audio recording* *Do you agree to take part in this interview and to have it recorded?* *Okay, first of all, thank you very much for allowing us to interview you* *To start the conversation on a more open note, let’s begin with:* *“How are you doing now?” and “How did you experience the rehabilitation process?”*.**		2 min
Topic list:	1. Goal/objective from hospital to geriatric rehabilitation	Did you have clear goals for what you wanted to achieve during your stay in the rehabilitation facility? * What were your specific goals?* Why did you choose those particular goals? Did you already have an idea of how you could achieve those goals? If yes, how? * **Follow-up questions:** Why is it important for you to achieve that goal? When you mention *being independent* or *returning to your previous level*, what does that mean to you? Can you give some examples?* When do you expect to be able to reach your previous level ?*	5 min
	2. Goals during the rehabilitation program in the nursing home	Did you have an intake interview? What goals did you discuss with the healthcare professionals?* Did you have any input in creating the treatment plan?* What was your contribution?* How did the intake interview go? How did you discuss your goals?*	3 min
	3. Working on goals during your stay in the nursing home	Who was involved in your rehabilitation process? What guidance did you receive to help achieve your goals? *(Alternative question: How were you supported in achieving your goals?)* What kind of guidance would you have wanted or needed?* What did you think of the frequency of contact with the therapists or healthcare professionals?* What helped you achieve your goals? What else would you have wished for to help you achieve your goals? Did you have to adjust your goals? If yes, why? To what extent have you returned to your previous level?	7 min
	4. Discharge to home	To what extent are you satisfied with the results you achieved?* What do you still need?* What kind of help did you need? What help would you still like now? What kind of guidance would you have wanted or needed to go home?* What helped you reach your current level? What has prevented you from reaching your desired level (yet)? Do you have any advice for us, or is there anything else you would like to share?	5 min
